# Cases of Poisoning by Opium

**Published:** 1846-05

**Authors:** Edmund Sharkey

**Affiliations:** St. Helier’s Jersey


*Cases of Poisoning by Opium.*—By EDMUND SHARKEY, M. D., St. Helier's Jersey. April 18, 1845.—Mr. Adolphus Von Kummer, a foreigner for some months resident here, was found dead in his bed this morning. The following particulars appeared in evidence before the Coroner's jury. He had been labouring under pecuniary embarrassments lately. Yesterday morning, after breakfasting in a hotel, and (as appeared from circumstances detailed in evidence) there taking a large dose of laudanum, he retired into a bath-room of the public baths, and after a lapse of about two hours was found there in a lethargic condition, from which, however, he was roused by the application of cold napkins to his head, shaking him, &c. About 10 o'clock that night he was visited by Dr. King, who remained with him for twenty minutes, during which time he conversed rationally, and seemed quite collected. Dr. K. left him. After Dr. K.'s departure he bathed his feet, and had a bowl of gruel brought to him. At 5 A. M. to-day Dr. K. was summoned to see him, and when he arrived found him comatose, and he died about 8 o'clock A. M. The servant had heard him snoring loudly in the night. Drs. King and Le Cocq, with myself, examined the body within twelve hours after death. The following appearances were noted :—

A quantity of white froth surrounded the mouth and nose. Body still warm and rigid. Stomach healthy; containing a quantity of fluid, apparently gruel, which had not any smell of opium. Intestinal canal also healthy, as were all the other viscera. Head: veins of scalp gorged with blood, also the veins of dura mater, sinuses, &c. There is no effusion in any part of the brain. The contents of the stomach were tried by the usual tests, but exhibited no trace of morphia or meconic acid. However, from all the circumstances of the case, we could come to no other conclusion than that he had died of the narcotic effect of some drug, which must have been taken subsequently to Dr. King's visit on the night preceding his death; and yet no phial, box, or paper was found. But on raking out the cinders of his fire-place a small mass of melted glass was discovered; so that probably, after drinking the contents, he threw the phial into the fire. It was proved in evidence that two separate purchases of half an ounce of laudanum each were made at the shops of two different druggists.

April 19, 1845.—Philip Mourant, æt. 32, an able-bodied sailor, had been for some little time under treatment for an apparently slight febrile attack, from which he was so far convalescent yesterday as to have gone to put his clothes on board the vessel in which he intended to have sailed on the day following. In the course of yesterday evening, after a hearty meal, he was attacked by a severe pain in his back, for which one of the company recommended him to take a dose of opium. A chemist was accordingly applied to, who gave three pills, containing each about one grain and a quarter of Extract. Opii. He took two of these pills, and was immediately afterwards attacked by a convulsive fit, and died. This evening, in company with Dr. Hooper, who had attended him during his late indisposition, I examined the body. The following particulars were noted.

He was a most muscular man, very fat, short-necked, and plethoric. There was an extensive lividity of the integuments of neck. On opening the head, a great quantity of blood flowed from the scalp, superficial veins, and sinuses of the brain. Some effused blood in a fluid state, and a small clot, were found surrounding the medulla oblongata. Stomach contained a quantity of fluid of a reddish tinge, probably from admixture of some wine which he had taken. Extensive ecchymosed patches were observed on its mucous membrane. Gall-bladder distended with bile. Heart flaccid and pale, nearly empty, and *when handled crepitates distinctly like a piece of lung*; on dividing the large vessels, a small quantity of fluid blood escapes, containing a great deal of gas. Fermentation seemed to have commenced in the stomach, as many bubbles escaped in the fluid which issued at the nostrils on pressing the epigastrium. Vena cava and venous system generally much gorged. These cases present a few points of interest in a medico-legal view. In the first we may observe the rapidity with which all traces of opium had vanished from the stomach, eluding not only the sense of smell, but also delicate chemical tests, so that, if it had not appeared by other evidence that the poison had been taken, and that in large quantity too, the medical witnesses could not have made any positive declaration about it. Again, the recovery of the suicide from the effects of the first dose, and his death so soon after, would have been rather embarrassing, had not the additional circumstances of the melted phial thrown an auxiliary light on the medical probabilities in the case. This affords another proof of the necessity of closely scrutinizing all the circumstances in every instance.

The second case is also rather a remarkable one, especially as regards the condition of the heart and the blood contained in it. The temperature at the time scarcely allows us to suppose that the contained gas could be the result solely of decomposition after the lapse of twenty-four hours; so that we are led to the conclusion that the blood must have been in a morbid condition, which predisposed to the fatal effusion. An important and difficult question was put to the medical witness by the jury, viz. "whether we thought that the dose of opium taken had accelerated the death." Now in any ordinary case, such a dose would perhaps have been innocuous. But if, as in the case before us, there was such a state of circulation as to cause a strong tendency to sanguineous effusion, it is undeniable that even such a dose would be hazardous; and it is quite certain that no judicious physician would think of prescribing opium *in any quantity* for such a patient. This was the substance of the answer which we gave.

Lastly, I think the perusal of both cases cannot but force upon our minds the conviction, that there is something needed in our medical police, as to the unrestricted dispensing of dangerous drugs by chemists and others, to the manifest danger of the community.

[REMARKS.—Although Dr. Sharkey's opinion that a second dose of opium was taken, in the first case, may be perfectly correct, and the deceased may have taken at different periods the two half ounces of laudanum which it was proved he had purchased, yet it may be proper to observe that several cases are on record, which show that a person may recover from the first symptoms, and yet ultimately die from the effects of a single dose.—*Ib.*

HOMŒOPATHY, MESMERISM, ETC.

The following remarks, which we extract from the *Epoque*, were made by M. Magendie, on opening his course of physiology at the College de France:

I have long been in the habit, previous to resuming my lectures, of devoting a few minutes to an inquiry into the actual state of medicine, and to an examination of the modifications which the theory and the practice of medical science may have undergone during the preceding year. This retrospective analysis will to-day furnish us with a certain number of reflections, the importance of which it would be in vain to endeavour to conceal, referring as they do both to the present and the future state of the profession.

Medicine and medical practitioners have attracted much attention during the last year. Many members of our profession have sat in congress, government has issued committees, the ministers have made solemn promises, and it is said that a law to regulate our destinies will shortly be presented to the Chambers. Everything, therefore, seems to smile upon us, and the wind swells our sails. But in the midst of these favourable circumstances—of these happy omens, alarming symptoms are discerned. The future prospects of medicine are the subject of deliberation, but ought we not sooner to deliberate on its very existence.

Medicine can only exist but inasmuch as patients have faith in it, and claim its assistance. It is not by theories that it lives, but by clients. Now it is impossible to conceal to ourselves that, at present, a certain proportion of the public abandons classical medicine, ironically called *old medicine*, and throws itself into the arms of new systems, thereby firmly believing that it associates itself to the progress of intellect.

Homœopathy—for it is more especially to it that I allude—aspires to nothing less than to overthrow the entire medical edifice with the arms of ridicule and contempt. And do you know how many specifics it numbers? Thee hundred and fifty! With a few globules of aconite, at the dose of a billionth of a grain, it produces the same effects as a bleeding of twelve or sixteen ounces. You exhaust your intellectual energies to find out the seat and the nature of a disease. Vain efforts! Homœopathy shows that every morbid symptom has its origin in psora, a kind of imponderable agent, which therapeutic substances reduced to unimaginable proportions alone can combat. Thus it is only with infinitely small means that infinitely large effects can be produced.

But, you will say, the persons who believe such absurdities are poor dupes, and the men who prey upon their weakness, hardened quacks. You are perhaps right; but listen to those whom you meet in society, and you will be surprised to hear of the wonderful cures that homœopathy has performed. No doubt its promises are often cruelly falsified, but the failures are but little spoken of. Moreover, we must not deny that many patients have recovered their health, in a most unhopedfor manner, whilst under homœopathic treatment. It is easy to understand that enthusiasm or the love of gain should take advantage of such facts to exalt the marvels of homœopathy. This brings us back to a question which I have often raised, and which I have endeavoured to elucidate by experiments for the last ten years—viz., what is the influence of treatment on the progress of disease?

In hospitals, as well as in private practice, we must first take into consideration the influence of the mind of the patient. Now there can be no doubt but that a patient who takes a medicine, experiences immediate benefit, from the conviction that it will favourably modify his disease. If this favourable result takes place, what has been the real share of the medicinal substance administered? Medical men are always inclined to attribute the cure of the disease they treat to the means which they have employed; but recollect that disease generally follows its course, without being influenced by the medication employed against it. Thus it is that you are often much deceived. A given medicine will succeed in an apparently serious case, and will fail in another case of a less dangerous character, without your being authorized to attribute to yourself in any way the success or the failure.

These reflections explain at once the cures of which homœopathy is so proud. Homœopathy, instead of bleeding a patient, will place gravely on his tongue a globule of aconite, which he will swallow with confidence and faith. You then see the disease improve. But it would have improved just as well without globules, provided some singular operation had struck the imagination of the patient. It really is too great a stretch of credulity to believe that a globule prepared by the formulæ of Hahnemann can contain any active principle. But, on the other hand, any one who has seen disease, must at once admit that this same globule may exercise, through the imagination, a powerful moral effect. You must not, indeed, accuse me of partiality towards homœopathy, when I state that I firmly believe that a physician would cure a patient sooner with globules, if the patient has faith in them, than with the most appropriate medicinal substances, if he distrusted their action.

What I state respecting medicinal substances is equally applicable to bleeding. A patient is seized with the symptoms to which the term inflammatory has been applied, and asks to be bled, believing that the loss of blood will cure him. You open a vein, and the abstraction of a certain quantity of the vital fluid is followed by an amelioration of the symptoms. But take care how you interpret the fact; the improvement may be owing to the moral effect produced, more than to the venesection. I will mention as a proof what I have often observed in my wards at the Hôtel Dieu. A patient labouring under acute disease—pneumonia, for instance—enters the hospital, believing firmly that he ought to be bled; I bleed him, but merely to the extent of two or three ounces; too small a quantity for the circulation to be in the least influenced by its abstraction. Nevertheless, the patient becomes more calm, and says he is better. A mere trial of bleeding will thus often suffice to arrest the progress of a disease which under another physician would be treated by abundant depletion. For more than ten years I have not found it necessary to have recourse to copious bleeding; in other words, I have rather endeavoured to act on the mind of the patient than on the circulation, and I have no hesitation in asserting that my practice has not been the less successful. Indeed, were I to tell you my mind entirely, I should say that it is more especially in the hospitals, in which the most active treatment is adopted, that the mortality is the most considerable. You must not, however, think that I wish to proscribe bleeding; I only wish to warn you against the deductions that may be drawn from its results.

After mentioning various other pseudo-medical delusions, M. Magendie continued: "It is with regret that I have laid before you this melancholy picture of the deplorable abuses which exist in the medical world; I am afraid no laws on the practice of medicine will be able to eradicate them. The quack will always contrive to elude the wisest regulations, for he knows that patients want to be deceived. Yes, gentlemen, we love error, and often refuse to yield to evidence, even when it is proved to us that our good faith and credulity have been imposed upon. Of this fact I will give you the following proof. A lady, a fervent believer in mesmerism, asked her niece, who was poorly, for a lock of her hair, in order to consult a somnambulist. The niece, wishing to try the credulity of her aunt, gave her the hair of her maid instead of her own. A renowned somnambulist was consulted, and at once recognised, by the hair of the maid, all the symptoms presented by the niece, whose sufferings she minutely described, to the great edification of the lady. The latter was then informed of the trick that had been played. You would naturally have thought that she would have recognised the imposture of the somnambulist. Not at all; she preferred concluding that the maid servant had the same disease as her niece, and obliged her to submit to a regular treatment, as if she had been poorly, although at that time in the best possible health.

Thus, many causes contribute to throw uncertainty on the result of medical treatment, either rational or empirical. Sometimes it is the patient who will be deceived; sometimes it is the quack, who deceives knowingly; sometimes it is the conscientious physician, who, notwithstanding his honesty, allows himself to be led into error by an erroneous interpretation of the means employed.

In the presence of such difficulties, it is less desirable for us to endeavour to lay down some law, than to give to our studies a more serious direction. Instead of demonstrating to pupils theories already framed, and often very badly framed, it would be better to teach them rather to study facts, and to take as their guide experimental observation. We have seen the rise, and, in the course of a few years, the fall, of a system which rests entirely on a single undefined word—inflammation. This system, opposed to all physiological ideas, was erroneously called "physiological medicine;" and yet its author was neither devoid of talent nor of knowledge. Such, no doubt, will be the destiny of those pretended methods of treatment which are, in general, founded on visionary speculations, have fashion for support, and dupes for followers.

As for us, gentlemen, we shall continue, as we have hitherto done, to study phenomena in the organs in which they are accomplished. Although the part of the physician may not be always as efficacious or as active as we could wish, when disease requires our assistance, we may still, by well-judged intervention, assist Nature in overcoming the functional derangements which give rise to disease. We shall more especially, therefore, endeavour correctly to appreciate the physiological action of organs. How, in reality, can we have recourse to rational treatment, when we are ignorant of the normal conditions of the economy?"—*London Lancet.*

